# Editorial: Visibility matters – women in neonatology

**DOI:** 10.3389/fped.2022.1059426

**Published:** 2022-11-07

**Authors:** Erin V. McGillick, Camilla Gizzi, MaryAnn Volpe, Britt Nakstad, Elisabeth M. W. Kooi, Julie Wixey, Mary Tolcos

**Affiliations:** ^1^ Clinical and Health Sciences, University of South Australia, Adelaide, SA, Australia; ^2^ The Ritchie Centre, Hudson Institute of Medical Research, Melbourne, VIC, Australia; ^3^ The Department of Obstetrics and Gynaecology, Monash University, Melbourne, VIC, Australia; ^4^ Department of Neonatology and NICU, S. Eugenio Hospital, Rome, Italy; ^5^ Division of Newborn Medicine, Department of Pediatrics, Tufts Medical Center and Tufts University School of Medicine, Boston, MA, United States; ^6^ Division of Paediatric and Adolescent Medicine, Institute of Clinical Medicine, University of Oslo, Oslo, Norway; ^7^ Department of Paediatrics and Adolescent Health, University of Botswana, Gaborone, Botswana; ^8^ Division of Neonatology, Beatrix Children’s Hospital, University Medical Center Groningen, University of Groningen, Groningen, Netherlands; ^9^ Perinatal Research Centre, Centre for Clinical Research, Faculty of Medicine, The University of Queensland, Brisbane, QLD, Australia; ^10^ School of Health and Biomedical Sciences, RMIT University, Melbourne, VIC, Australia

**Keywords:** neonatology, gender equity, diversity & inclusion, clinical, preclinical

**Editorial on the Research Topic** Visibility matters – women in neonatology By McGillick EV, Gizzi C, Volpe MA, Nakstad B, Kooi EMW, Wixey J, Tolcos M. (2022) Front. Pediatr. 10: 1059426. doi: 10.3389/fped.2022.1059426

We are delighted to present the inaugural Frontiers in Pediatrics Women in Neonatology series. Gender inequity in health and medical research has been perpetuated by long-standing biases (including unconscious bias), stereotypes and structural barriers to the recruitment, retention, career progression and recognition of women's achievements. While many structural changes are required to address these factors towards achieving equity, active allyship and visibility is also important to support and encourage the next generation of women and girls to pursue research careers. This article collection includes 5 original research papers, 2 brief research reports and 1 review article demonstrating a diversity of neonatology-related research led by women.

Depicolzuane et al. reviewed and presented a summary of animal and translational studies, and clinical observations on the role of lung surfactant protein A in lung function and importantly in lung protection from both infectious and non-infectious agents. Surfactant protein A has a unique role in the lung through its interactions with lung macrophages where it impacts the activity of alveolar macrophages in response to injurious agents or stimuli. Interestingly, surfactant protein A also has a role in control of the *in utero* environment.

In a mixed methods study, Eidger et al. investigated the perceptions of, and barriers to, effective teamwork within the multi-disciplinary context of neonatal resuscitation. The study explored the interconnected themes of team composition, hierarchy, leadership, communication and training. They highlighted the importance of team environments that value diversity of individuals training and experience, active leadership, effective communication, and modelling of supportive environments that enable all team members to feel comfortable speaking up.

In the neonatal intensive care unit (NICU), mothers are at risk of developing maternal distress and depression. Mautner et al. analyzed questionnaires on postnatal depression and resilience from mothers of term and preterm babies admitted to the NICU. The mothers with high resilience experienced significantly less depression compared to the mothers with low resilience scores. The authors suggest that evaluation of resilience should be included in standardized psychological follow-up enabling mothers to develop personalized strategies to cope with the distressing situation of having a child in the NICU. Further, social support from the partners, family members, friends and health care providers should be facilitated.

Preterm infants are at a higher risk of adverse neurodevelopmental outcomes than infants born at term. As an adverse fetal environment may play a key role in these outcomes, Van Dokkum et al. investigated potential underlying mechanisms of neurodevelopmental impairments using placental tissue. The study assessed whether placental DNA methylation of several genes affected early neurological functioning in preterm infants. In a cohort of 43 infants, the authors showed hypomethylation of *NR3C1* (gene encoding for the glucocorticoid receptor) in placental tissue to be associated with poorer neurological outcomes at 3 months of age.

Helguera-Repetto et al. described infant seropositivity in the context of maternal SARS-CoV-2 infection and infant outcomes. This retrospective nested case-control study enrolled pregnant women with a positive SARS-CoV-2 RT-PCR test and their term infants. SARS-CoV-2 antibodies were present in 76% of neonates from seropositive mothers with a positive association between maternal IgG concentration and cycle threshold values with infant antibodies. Most neonates were asymptomatic, but 14% of seronegative neonates presented with respiratory disease compared to non-seropositive neonates. In addition, the odds of infant respiratory morbidity tended to decrease when infant IgG levels increased.

In a retrospective study, Mulot et al. reviewed infants <34 weeks gestation, hospitalized before 12 months due to a confirmed Respiratory Syncytial Virus (RSV)-related lower respiratory tract infection (LRTI). LRTI was considered very severe (VS-LRTI) when patients needed positive pressure ventilation. The main risk factor for VS-LRTI was a younger age corrected for prematurity at the onset of infection. Surprisingly, infants with VS-LRTI had higher gestational age and birthweight, a lower incidence of bronchopulmonary dysplasia and attended community childcare less often. Efforts to better understand risk factors associated with severity of RSV infections are necessary to elaborate early intervention strategies and monitoring plans.

In a preclinical study by Forbes et al., mRNA expression of *TRPM6* and *TRPM7*, transmembrane cation channels thought to regulate intracellular cardiac calcium in the neonatal period, was examined in the preterm and term piglet heart; the effect of sex, antenatal glucocorticoids and the transition to postnatal life was also assessed. Increased *TRPM7* mRNA in the piglet heart is a mature response to term birth, but is absent in preterm piglets, particularly those that did not receive antenatal glucocorticoids. Interestingly, *TRMP7* mRNA was reduced in male preterm piglets. If this reduction limits myocardial calcium handling, this finding might contribute to the poorer outcomes for male preterm infants. The authors propose that targeting TRMP7 might improve cardiac function in preterm infants.

In a retrospective single centre study, Xodo et al. investigated how postnatal outcomes differ between preterm infants exposed to single dose or complete course of antenatal glucocorticoids. A complete course of antenatal glucocorticoids has significant clinical benefits for preterm infants including decreased respiratory morbidity and mortality, decreased neurological complications, improved maturation, and improved neurodevelopmental outcomes. Unfortunately, preterm infants often need to be delivered prior to a complete steroid course. This cohort showed that infants receiving one dose of antenatal glucocorticoids had an increased incidence of severe grades of intraventricular haemorrhage, periventricular leukomalacia, and worse respiratory course, conditions that impact neurodevelopmental outcome.

This article collection highlights work led by women in neonatology and their teams across 8 countries, including 64 authors spanning 39 clinical and research departments. It presents advances in theory, experiment, and methodology with applications to critical problems in neonatology, supporting healthcare teams and parents. In addition to presenting diverse research topics, it also demonstrates a breadth of diversity and intersectionality of the neonatology research community. As this Frontiers in Paediatrics series grows to support gender equity, we must continue to address inequities facing women, non-binary and gender diverse clinicians and researchers. Our community is richer and possibilities greater when we each actively advocate for and work towards equitable, diverse, and inclusive environments in the clinic, our research and teams.

